# Comparative and Combinative Study of Urban Heat island in Wuhan City with Remote Sensing and CFD Simulation

**DOI:** 10.3390/s8106692

**Published:** 2008-10-25

**Authors:** Kun Li, Zhuang Yu

**Affiliations:** 1 School of Architecture, Tsinghua University, Beijing 100084, P.R. China.; 2 Architecture & Urban Planning College, Huazhong University of Science and Technology, Wuhan 430074, P.R. China.

**Keywords:** Urban Heat island, Urban Planning, Remote sensing, Computational Fluid Dynamics, Wuhan

## Abstract

Urban heat islands are one of the most critical urban environment heat problems. Landsat ETM+ satellite data were used to investigate the land surface temperature and underlying surface indices such as NDVI and NDBI. A comparative study of the urban heat environment at different scales, times and locations was done to verify the heat island characteristics. Since remote sensing technology has limitations for dynamic flow analysis in the study of urban spaces, a CFD simulation was used to validate the improvement of the heat environment in a city by means of wind. CFD technology has its own shortcomings in parameter setting and verification, while RS technology is helpful to remedy this. The city of Wuhan and its climatological condition of being hot in summer and cold in winter were chosen to verify the comparative and combinative application of RS with CFD in studying the urban heat island.

## 1. Introduction

Nowadays, one of the critical problems of cities in China is the tendency in city development towards maximization and urbanization. The local characteristics, such as climate and ecological environment, are often neglected in urban planning. These phenomena boost the effects of heat islands. Cities need reasonable planning and utilization of ecological resources to relieve urban heat islands, but traditional urban planners have no suitable methods to solve this problem, as they may have no idea of how to build relationships between urban planning and the mitigation of heat island effects. Therefore a comparative and combinative digital research method is presented here to provide advice for planners, so they can deal with heat environment problems based on scientific methods. In another way, a city is huge and complicated and isolated research on city problems can hardly achieve reasonable results. Collaboration between different technologies and disciplinary fields can help to detect the intrinsic causes of a city's problems from different points of view, so this synthetical research mode includes several digital methods such as RS and CFD technology which are combined to investigate heat islands and heat environments based on the usage of natural resource and the level of urban planning.

There is considerable relevant research on urban heat islands. Stathopoulou *et al.* used ETM images to analyze the urban heat islands of major cities in Greece during the daytime [1]. Rizwan *et al.* concluded that the heat re-radiated by urban structures plays the most important role in urban heat islands [2]. Jusuf *et al.* studied the influence of land use on the urban heat island in Singapore [3]. Fei Yuan *et al.* compared the normalized difference vegetation index (NDVI) and impervious surface area as indicators of surface urban heat island effects in Landsat imagery [4]. Mochida *et al.* investigated the effects of urbanization on heat island circulation over the Tokyo area by a CFD method [5]. Ashie *et al.* developed a building canopy model coupled with CFD and discussed the effects of building planting for the decrease of urban heat island phenomena and energy consumption for cooling [6]. These researches made great contributions to the study of urban heat islands. In this work, our study is focused on urban planning, and collaboratively uses the advanced digital approaches of remote sensing along with computational fluid dynamics to do a comprehensive analysis, and uses Wuhan city – an area that is hot in summer and cold in winter, as a case study. Both the current status of the heat environment in Wuhan and the corresponding improved measurements at the urban planning level are tackled by comparative and combinative methods.

Precision is the main problem in city research. The subject of urban planning is characteristically illegible and indeterminate. The research field is more concerned about prediction and collective effects, so urban planning's requirement for precise digital methods is not very high.

## 2. Methodology

### 2.1. Calculation of inversion model in Remote Sensing

Thermal infrared inversion technology in Remote Sensing can provide the distributed characteristics of urban heat islands. Thermal infrared imaging techniques can turn the invisible thermal radiation distribution on an object's surface into visualized thermal images [7]. Our evaluation of land surface temperatures uses ETM satellite images and the inversion model of the mono-window algorithm [8]. The thermal infrared band of ETM is used to calculate land brightness temperatures and the meteorological data of temperature, humidity and so on are used to revise the land brightness temperature to obtain the land surface temperature (LST) [9]. The calculation formula is shown below [10]:

(1) $$\text{Ts}=\left[67.3554*(\text{C}+\text{D}-1)+(0.4414*(\text{C}+\text{D})+0.4586)*{\text{T}}_{6}-\text{D}*{\text{T}}_{\text{a}}\right]/\text{C}$$

where the unit of T_S_ is K; T_6_ is land brightness temperature. The values of C and D are calculated as follows [11]:

(2) C=ɛτ

(3) D=(1-τ)*[1+(1-ɛ)τ]

where ε is surface emissivity and τ is atmospheric transmittance.

### 2.2 Computational Fluid Dynamics

Computational Fluid Dynamics is an effective simulation technology to analyze flow motion and can deal adequately with most flow fields [12]. The urban heat environment can be studied using this technology to simulate heat flow. It is assumed that the city lies in a flow field generated by atmospheric motions and the main form of air flow is wind. A digital model of a city is set up and is then put through a computer-aided wind tunnel simulation. Various conditions of climate and regions are turned into boundary condition settings suitable for computer processing. Finally, measurements and CFD equations are used to deal with the atmospheric flow in the city and urban districts [13]. The temperature distribution, heat island conditions and so on are calculated, and the effects of certain urban planning measures can be examined in the CFD simulation. CFD technology thus represents one of the main methods for the research of urban heat environments.

These two technologies are joined together to study the heat environment. Remote sensing mainly focuses on the condition of the whole urban heat environment and gives the macroscopic evaluation criterion of heat environment, while CFD technology mainly focuses on the running status and space dynamics of heat environment and the heat transfer function of wind in heat flow under the influence of the urban climate and underlying surfaces.

## 3. The case study of Wuhan

Using the above-mentioned methods, the city of Wuhan in China was chosen to do the urban heat island case study. It is a typical Chinese city, being hot in summer and cold in winter. It lies in the north subtropical monsoon area (E113°41′∼115°05′, N29°58′∼31°22′) and thus belongs to the subtropical humid monsoon climate region, with an abundance of rain and sweltering heat [14]. Wuhan city has the worst climate conditions of any similar latitude areas on Earth, with extremely high temperatures, for which it is known as the “Furnace City” [15].

### 3.1 Comparative study of urban heat island using RS technology

The data used was the ETM satellite image on July 9^th^, 2002, being retrieved from the *Data-sharing Network of Earth System Science* (www.geodata.cn; all the ETM/TM satellite images used in this research were acquired from this website, except when specially mentioned.). The land surface temperature of Wuhan city at the time the satellite passed by was calculated, then the relevant ETM bands were used to do the classification and obtain corresponding a surface classification map. The whole city is divided into eight regions: high density; sub-high density; middle density; low density; vegetation; rivers; lakes and “other”. Then the relevant heat island research can be done. This paper focuses on the application of RS technology and mainly pays attention to the use of the RS results in urban planning. Many factors will influence the RS reversed result, but they are not the main aim of this paper and are not discussed in detail.

#### 3.1.1 Different scale comparison of heat environment situation in Wuhan

The terrain classification results use pixel units. Since the terrain classification needs to use the previous seven ETM satellite image bands, all the bands' resolution should be resampled to 30 m, which means the surface area of one pixel is equivalent to 30 m x 30 m. The statistical analysis of image classification is carried out to get the mean value, extreme value and mean square deviation of a certain terrain, and index information for the various terrains is stacked together to analyze the relationship between heat island and terrains by means of the statistical analysis of the terrain classification image. The information images which should be stacked include: land surface temperature image, NDVI image, NDBI image and so on. Wuhan city is divided into two scale regions: large scale, including the whole city area; and small scale, including only the downtown area of city. Then the situation summarized in Tables 1 and 2 is obtained.

It can be seen from Table 1 that the underlying surfaces of Wuhan city mainly include city impervious surfaces, vegetation surfaces and water surfaces. Each of them occupies about one third of the area.

The degree of greening in Wuhan is relatively high on the big scale, and its greening ratio reaches 30%, but the distribution of underlying surfaces is unreasonable. In the downtown area (small scale), vegetation surfaces occupy a very small ratio, only reaching 18.27%, with most of them lying in suburban areas. From the examination of the satellite images, the vegetation in the main region of the city was reduced significantly, being mainly composed of small green blocks, scattered in natural resource points and parks in the city, while high density building regions in city have much less green spaces.

Water resources are abundant and large rivers and lakes exist in every city region. On the other hand, semi-dry regions, like wetlands, also occupy large areas. Many of these surfaces are shoals, fishponds and so on, coming from the rivers or lakes. Even though they are not complete water bodies, they also perform the function of adjusting urban climate and produce masses of fresh air. They are called the “green lung” of the city. They also need to be protected because these regions have high ecological fragility, being apt to break the ecological balance.

City impervious surfaces are mainly composed of buildings, streets and so on and are aggregation places of city living. These places have high population, masses of motor vehicles and high density buildings, so the anthropogenic heat of these places is intense. These places belong to reinforced concrete underlying surface, giving high radiation temperatures, so the land surface temperature of impervious surfaces is very high, being the most centralized places of the heat island. The higher the building density, the more serious is the heat island. Land surface temperature image shows that the regions of high land surface temperature lie mostly in the city impervious surface.

#### 3.1.2 Urban heat island comparison at different stages

Compared with the situation of July 9^th^ in 2002 mentioned above, the TM image of July 19^th^, 1991 (acquired from Global Land Cover Facility of University of Maryland: http://glcf.umiacs.umd.edu/data/) was used to study the influence of city development on urban heat environment.

The city area of Wuhan in 1991 was much smaller and building density was also lower. On the contrary, the NDVI index in 1991 was much higher than that of 2002. The minimal NDVI index of main city region in 1991 was -0.541, its maximal value was 0.699 and the mean value was 0.046. The minimal NDVI index in July 9^th^, 2002 was -0.964, the maximal value was 0.640 and the mean value was -0.169. For the further study, 16 typical regions are selected from satellite images to do the comparison of NDVI index and land surface temperature between these two periods. These typical regions represent various surfaces in city. Table 3 shows the results.

**Figure 1.**
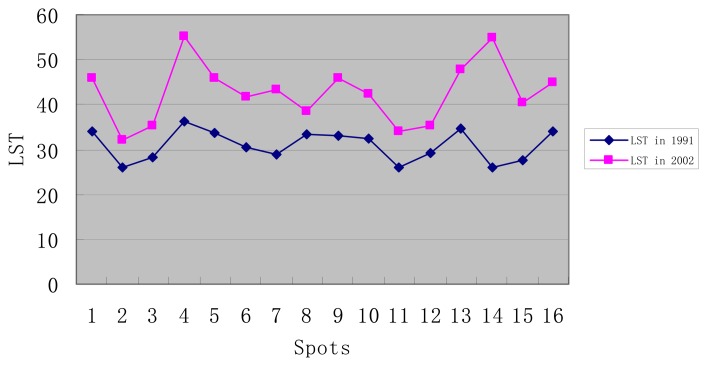
Land surface temperature (LST) comparison between July 19^th^, 1991 and July 9^th^, 2002.

**Figure 2.**
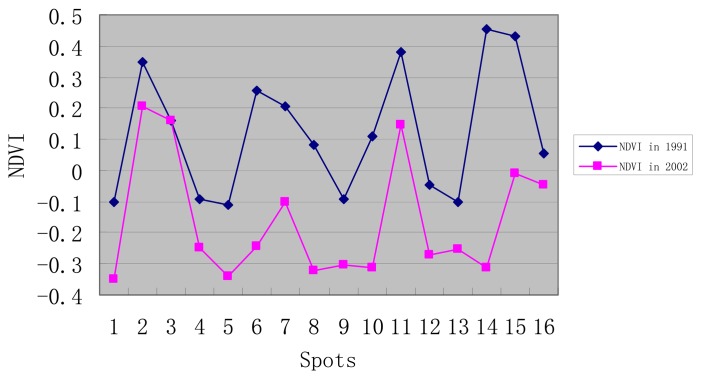
NDVI comparison between July 19^th^, 1991 and July 9^th^, 2002.

Meteorological data show that the temperature on July 19^th^, 1991 was 33.9°C and relative humidity was 70%, while the temperature on July 9^th^, 2002 was 29.2°C and the relative humidity was 56%, so it can be seen that these two days had similar and comparable climate conditions. From Figures 1 and 2, conclusions may be drawn as follows:

The vegetation status of Wuhan in 1991 was better than that of 2002 and every selected points' NDVI were higher than those of 2002. The vegetation cover in 1991, whose NDVI value was up to 0, was larger than that of 2002. Land surface temperature of each point in 1991 was lower than that of 2002. However, the urban heat environment structure in 1991 was close to that of 2002. The most serious urban heat island regions in 2002 were also the regions with the highest land surface temperature in 1991. The major heat island contributors were factories. The next were old local regions, commercial streets and traffic architectures, such as railway stations. The third ones were residential areas. The last were low density administrative buildings, like schools, city gardens and public green spaces. The images from the two dates show the same conditions.

A heat island already existed in Wuhan in 1991, but the temperature difference between the interior and exterior of city was not so large. The urban heat environment was comparatively comfortable and no region in the city had radically high land surface temperatures.

The old city zone had good climate adaptability in 1991, although the building volume rate was high. The streets in these regions followed the prevailing wind direction and ventilation was good. However, building density by 2002 had risen rapidly and the building volume rate grew much higher in central city. Highrise buildings around the old city zones blocked their ventilation passages, and many old city zones were demolished and renovated, thus changing the characteristics of underlying surface, so old city zones became high temperature zones where the land surface temperatures were all above 45°C.

City green spaces correspond to the low land surface temperature zones in the city and play the main role in adjusting the city temperature. Land surface temperature of city gardens in 1991 was less than 30°C and vegetation coverage was high. However, NDVI index in city garden in 2002 was greatly reduced and the land surface temperature rose significantly. Though they still kept a low level of land surface temperature in the city, their temperature regulating function has been dramatically reduced. This is due to the fact that the vegetation inside city was not protected effectively. Thus, building density and vegetation coverage should be kept in a proper range of proportion in urban planning.

City temperature field is an integrated whole and changing land surface temperature around a region will influence the region's land surface temperature, even its own condition doesn't change. Zhongshan garden in central city of Wuhan may be a good example. Its NDVI index was 0.16 in 1991 and 0.159 in 2002. Although vegetation coverage almost didn't change, its land surface temperature turned from 28.26°C to 35.43°C, so protecting a city's green gardens alone is not enough, because land surface temperature still could rise, even if NDVI of a local region remains stable.

#### 3.1.3 Comparative study of heat environment with another typical city

Shanghai city, located at the seaport of Yangtze River and lying at the most eastern part within this zone, is another typical city, being hot in summer and cold in winter. The land surface temperature of Shanghai was retrieved and relevant heat island indices are deduced. Compared with the situation of Wuhan city, the common heat environment features in this zone are as follows:

The regions along the Yangtze River in the cities studied are the most obvious urban heat island places. It is the important commercial development region for its good environment and shipping superiority, so the riverfront region has the highest density of buildings and population.

Urban heat island in the factory region is the strongest. The next is the commercial region and residential region of relatively high density. Last is the city region of normal density. Vegetation alleviates the heat island effect. The more the vegetation covers, the weaker the heat island affects. Heat islands are kept low where the regions have large ecological resource areas. It can be seen that Wuhan city and Shanghai city have similar structure and distribution of heat island and the above-mentioned heat environment analysis has universality.

### 3.2 Heat environment study for city development planning with joint use of CFD simulation

The above sections have introduced the study of urban heat environments with RS technology. Its shortcomings are: no flow field analysis and no optimization design. CFD technology can deal with these problems, as it is able to do flow analysis and optimization design during the study of the heat environment of cities and city regions. CFD technology has its own problems, such as difficulties in parameter setting and verification, which can be remedied by adopting relevant parameters and results of RS technology.

The working process of CFD simulation includes several steps: simplifying the city regional diagram, creating digital models, setting boundary conditions, and carrying out model calculations. Various parameter settings are difficult, yet crucial to CFD simulation. Each city's conditions, including building density level, ecological resources and climatic situation should be materialized through parameter settings. It is necessary and advantageous to introduce RS technology into parameter settings in each step of CFD simulation. Of course, CFD simulation and RS technology use different types of methods to depict urban heat island. Their combinations are focus on parameter settings and tendency comparison. Many RS indices reflect the characteristics of city surface, atmospheric conditions and so on, which can be used in CFD simulation.

**Figure 3.**
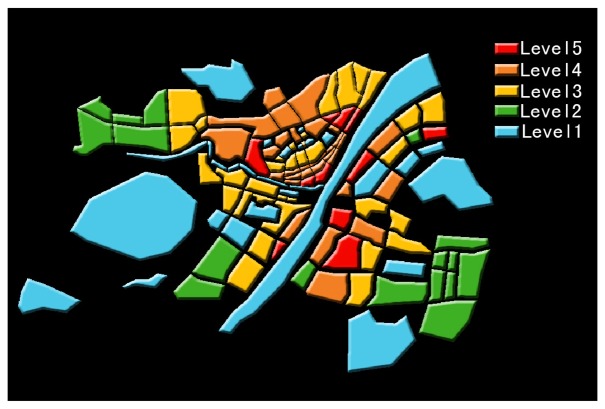
City region classification Based on land surface temperature from RS.

City grading according to the conditions of different city zones such as real building combination and vegetation terrain is an important parameter setting. Traditional city grading is based on experience and has strong subjectivity [16]. However, referring to the land surface temperature image of RS technology, city blocks can be subdivided into different grades according to the heat island level classified by land surface temperature and different boundary conditions are assigned accordingly. These may raise the simulation accuracy to reflect the real structure of urban heat island. Figure 3 shows the city level setting made by the above idea and the levels from five to one show the classification of underlying surface from nuclear heat island region to natural surfaces of gardens and water.

**Figure 4.**
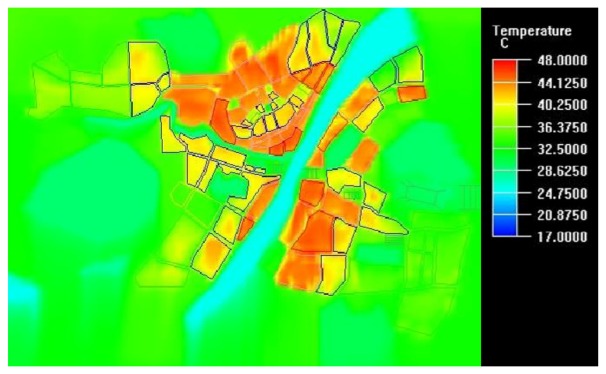
City heat environment simulation of Wuhan city.

Then the indices of surface emissivity, roughness and so on acquired from the deduction of ETM image are used in the block setting, making CFD simulation conforming to the features of city underlying surface. Utilizing the above-mentioned various settings, a high qualify CFD simulation resulted. The relevant simulation results include temperature image, wind speed image, wind pressure image and so on. These images not only predict the temperature distribution of the heat island, but also show the direction and ventilation effect of wind in different city regions. Figure 4 shows the simulation of city heat flow following the main wind direction. Comparing Figure 4 with the land surface temperature image, RS technology and CFD simulation reach high consistency in statistical deduction. Although the images have different meanings, they have same tendency in the expression of heat island distribution. From the image, the heat island situation and heat distribution are manifested clearly and these results are the same for the revised land surface temperature based on Remote Sensing. From this comparison, the accuracy of CFD is verified. The advantages of CFD simulation are not only reflecting the situation of temperature, but also revealing the running status of heat flow, such as heat transfer. All these aspects should be covered in heat island study. Otherwise, evaluation of the heat environment of a region is not complete and even prone to error. It can be seen from CFD simulation image that temperature between city and vegetation places at suburb changes linearly and gradually, like comet tail. The orbit's dynamic characteristics show that wind is the main cause of transferring the low temperature of natural surface in suburb to the city. Wind speed slows down when it is close to city and the decreasing amplitude of temperature also slows down, predicting that the temperature change from natural vegetation body to city is a continuous process with slow variation. Due to the same wind, the distribution of heat environment in city does not vary sharply and the temperature at the nuclear heat island turns to that of suburb by the diffusion tendency. The temperature around the Yangtse River is relatively high from the land surface temperature image display, but the temperature in these places is somehow lower than that of the places of the same parameter setting in CFD simulation, indicating that the adjusting capability of the Yangtse River is good, comparing the bad result made by RS analysis. Too high building density beside the Yangtse River is the real reason behind the high heat island. CFD simulation can reflect the ongoing status of urban heat environments well. City ventilation is poor and cannot dissipate heat well. Of course, conceptual and prospective tests can be done by CFD simulation to verify the feasibility of urban planning from the heat environment point of view and is not discussed here in detail.

## 4. Conclusions

Thermal infrared inversion technique of RS technology can be used to do rigorous analysis on urban heat environment. Comparative analysis of heat environment of different scales, stages and places is done using temporal and spatial analysis of RS technology. Dynamic heat flow analysis can be carried out with CFD simulation. RS technology and CFD simulation are mutually complementary and their shortcomings can be mutually remedied. All these comparative and combinative studies make accurate heat environment research much closer to reality. The urban heat environment is a systematic and comprehensive problem and requires digital and advanced research methods to obtain reasonable results.

## Figures and Tables

**Table 1.** Index of underlying surface of the big scale region of Wuhan.

**Region**	**Meters ²**	**Percentage (%)**	**Mean land surface temperature value (°C)**	**Mean value of NDVI**	**Mean value of NDBI**
High density	31,257,004.5	2.461	45.180924	-0.250509	0.263994
Sub-high density	77,053,284	6.067	43.405907	-0.245984	0.229854
Middle density	164,602,462.5	12.961	39.613286	-0.186561	0.179671
Low density	109,859,249.3	8.651	37.619073	-0.095846	0.151695
Vegetation	389,091,305.3	30.638	32.560922	0.20666	-0.023167
River	105,008,492.3	8.269	26.149353	-0.532438	-0.139054
Lake	107,268,171.8	8.447	28.83498	-0.41505	-0.110941
Other	285,804,783	22.505	32.34296	-0.163481	-0.025443

**Table 2.** Index of underlying surface of the small scale region of Wuhan.

**Region**	**Meters²**	**Percentage (%)**	**Mean land surface temperature value (°C)**	**Mean value of NDVI**	**Mean value of NDBI**
High density	24,326,887.50	3.238	45.430794	-0.253232	0.272563
Sub-high density	61,799,229.00	8.225	43.521017	-0.247469	0.235672
Middle density	106,861,234.50	14.222	39.955588	-0.194577	0.187942
Low density	51,787,435.50	6.892	37.800995	-0.093743	0.1439
Vegetation	94,657,990.50	12.598	32.856176	0.197542	-0.020667
River	48,274,454.25	6.425	25.926209	-0.537256	-0.121711
Lake	44,042,631.75	5.862	28.526494	-0.416726	-0.095614
Other	86,394,971.25	11.498	32.344091	-0.171347	-0.005489

**Table 3.** Comparison of indices of land surface spots.

**Name**	**Land surface pattern**	**Land surface temperature in July 19^th^, 1991 (°C)**	**NDVI on July 19^th^, 1991**	**Land surface temperature on July 9^th^, 2002 (°C)**	**NDVI on July 9^th^, 2002**
Hanzheng street	Commercial street	33.873688	-0.1	45.816256	-0.350427
Jiefang park	Green park	26.13504	0.348837	32.089508	0.204301
Zhongshan park	Green park	28.264862	0.16129	35.433502	0.159664
Wuhan steel plant	Factory	36.295788	-0.090909	55.130981	-0.25
Wuchang railway station	Traffic architecture	33.845154	-0.111111	45.852692	-0.341772
Guanggu square	Commercial building	30.487366	0.257732	41.7742	-0.244186
Xudong square	Commercial building	28.991302	0.207547	43.166731	-0.101604
Wuhan square	Commercial building	33.25412	0.08046	38.343536	-0.320388
Wenhua lane	Old city region	33.091064	-0.090909	46.032379	-0.302632
Jiqin Street	Old city region	32.419464	0.111111	42.206299	-0.314286
Guishan TV. tower	Green park	26.043304	0.37931	33.854095	0.146853
Qintai street	Traffic node	29.112122	-0.045455	35.436462	-0.270073
Yingwu state	Normal city region	34.66684	-0.103448	47.785339	-0.251613
Dongfeng stamping company	Factory	25.8414	0.452991	54.78064	-0.312977
Huazhong University of Science & Technology	University	27.517944	0.431818	40.284821	-0.008
Wuhan heavy hanging wall machine tool plant	Factory	34.070038	0.052632	44.867493	-0.045455
